# Ready to run the wards? – A descriptive follow-up study assessing future doctors’ clinical skills

**DOI:** 10.1186/s12909-018-1370-4

**Published:** 2018-11-12

**Authors:** Till Johannes Bugaj, Christoph Nikendei, Jan Benedikt Groener, Jan Stiepak, Julia Huber, Andreas Möltner, Wolfgang Herzog, Ansgar Koechel

**Affiliations:** 1 Department of General Internal Medicine and Psychosomatics, University of Heidelberg, Medical Hospital, Im Neuenheimer Feld 410, 69120 Heidelberg, Germany; 2 Department of Endocrinology and Clinical Chemistry, University of Heidelberg, Medical Hospital, Heidelberg, Germany; 3 Department of Cardiology, Angiology, Pneumology, University of Heidelberg, Medical Hospital, Heidelberg, Germany; 4 Center of Excellence in Medical Assessment, Faculty of Medicine, University of Heidelberg, Heidelberg, Germany; 5 Department of Dermatology, University of Tübingen, Medical Centre, Tübingen, Germany

**Keywords:** Assessment methods, Workplace learning, Clinical competencies, Internal Medicine, Final year medical education

## Abstract

**Background:**

Recent studies have shown that clinical tasks only represent a small percentage in the scope of final-year medical students’ activities and often lack sufficient supervision. It appears that final-year medical students are frequently deployed to perform “routine tasks” and show deficits in the performance of more complex activities. This study aimed to evaluate final-year students’ clinical performance in multiple impromptu clinical scenarios using video-based assessment.

**Methods:**

We assessed final-year medical students’ clinical performance in a prospective, descriptive, clinical follow-up study with 24 final-year medical students during their Internal Medicine rotation. Participating students were videotaped while practicing history taking, physical examination, IV cannulation, and case presentation at the beginning and end of their rotation. Clinical performance was rated by two independent, blinded video assessors using binary checklists, activity specific rating scales and a five-point global rating scale for clinical competence.

**Results:**

Students’ performance, assessed by the global rating scale for clinical competence, improved significantly during their rotation. However, their task performance was not rated as sufficient for independent practice in most cases. Analysis of average scores revealed that overall performance levels differed significantly, whereby average performance was better for less complex and more frequently performed activities.

**Conclusions:**

We were able to show that students’ performance levels differ significantly depending on the frequency and complexity of activities. Hence, to ensure adequate job preparedness for clinical practice, students need sufficiently supervised and comprehensive on-ward medical training.

## Background

On-ward team integration, independent patient management, and supervision are crucial facilitators for effective workplace learning and the successful acquisition of clinical competencies [1]. However, quantitative and qualitative studies on clinical rotations [2–6] have consistently revealed a severe lack of on-ward supervision, direct observation, and feedback as well as the unremitting assignment of final year medical students (FYMS) to non-instructive routine tasks. This fosters the impression that workplace learning still resembles rather a ‘black box’ approach than a well-organized learning environment and is sadly often a matter of trial and error [7].

Recent research by Bugaj et al. [8] suggests that FYMS’ assigned clinical tasks are mostly repetitive, of low-difficulty, and lack sufficient supervision. The study asked 34 FYMS to keep a detailed record of all their on-ward activities and to document the duration, mode of action, estimated relevance for later practice, as well as difficulty-level during their final-year Internal Medicine trimester. Drawing blood (20.8%) and full admission procedures (9.6%) were the most frequently actively performed medical activities, whereas ward rounds (42.0%) and meetings (29.6%) were the activities most often observed. 14.9% of the time was spent with nonmedical activities and 82.1% of all medical activities performed went unsupervised.

Following the basic principles of behavioral learning psychology, frequent repetition, independent practice, and personal responsibility lead to improved performance [9]. In return, more complex and comprehensive medical responsibilities, such as ward rounds, case presentations, and consultations make up a much smaller part of the students’ practical education and are, hence, presumably practiced less frequently and well. In addition to the described structural imbalance, it was reported that most of the daily tasks were performed without supervision. In a best-case scenario, this may result from professional entrustment, namely, based on an implicit evaluation of the students’ performance level, a senior clinician entrusts a task to a student after an adequate period of supervised observation. However, in reality, most tasks just seem to be ‘handed over’ without any prior performance monitoring or procedural controls [10], which may either be explained by the participating health care professionals’ poor working conditions and high workload or, from a more critical point of view, by “bad practice” [11, 12].

According to the study of Bugaj et al. FYMS are frequently assigned to “routine tasks” requiring little to no supervision, which might lead to pronounced deficits when it comes to more complex activities. In light of ubiquitous patient safety and quality management efforts, the question of securing performance levels sufficient to enable independent practice deserves careful consideration. Therefore, we aimed to assess FYMS’ clinical performance levels in multiple impromptu clinical scenarios using video-based assessment by means of four activities with different frequencies and different degrees of complexity: (I) IV cannulation (most frequently performed according to students’ self-estimation, low complexity), (II) history taking and (III) physical examination as parts of full inpatient admission (less frequently performed according to students’ self-estimation, more complex) and (IV) case presentation as part of active participation in ward rounds (rarely performed according to students’ self-estimation, high complexity). We hypothesized that FYMS’ performance-levels (i) are sufficient for unsupervised practice in those selected basic skills and activities that were extensively practiced in previous years at medical school, (ii) improve during Internal Medicine rotation, (iii) are higher for less complex and more frequently performed activities.

## Methods

### Design

We conducted a prospective, descriptive, clinical follow-up study to assess clinical procedural performance of FYMS. To this end, *n* = 24 FYMS deployed in the Department for Internal Medicine at the University of Heidelberg, Germany, were videotaped while performing four indispensable clinical activities (history taking, physical examination, case presentation, and IV cannulation – single execution of each activity) at two distinct points of time (t1 during the first and t2 during the last two weeks of the FYMS’ Internal Medicine rotation). Students’ performance was then evaluated independently by two blinded video assessors.

### Participants

All FYMS who enrolled in their Internal Medicine rotation in the Department for Internal Medicine at the Heidelberg University between May and September 2014 were invited to participate in the study on a voluntary basis. There were no exclusion criteria. Only one student declined participation (participation rate 96%).

### Patients

All participating patients were Internal Medicine inpatients at the Medical Hospital of the University of Heidelberg, Germany, and preferably stayed on the ward the final-year students were assigned to. Initial invitation, whenever possible, was given by the final-year student himself, while written consent was secured by the supervising physician.

### Assessment of FYMS’ baseline-characteristics

The assessment of FYMS’ baseline-characteristics included questions on age, sex, and career aspirations. Further evaluation focused on how often the students had so far independently performed the observed clinical activities during their studies in a) controlled conditions (i.e. skills lab, simulation and standardized patient training) or b) genuinely. The students gave estimations of how often they had performed each activity previously. Finally, students were asked to self-assess each of the four aforementioned clinical activities with regard to their feeling of job preparedness via a five-point Likert-scale (statement: *Concerning [activity named here] I feel well prepared for the job as a medical doctor*; 1 (*not true at all*), 2 *(not true)*, 3 *(undecided)*, 4 *(true)*, 5 *(very true))*.

### Accompanying FYMS curriculum

The study was embedded in our final-year medical curriculum [13] starting with interdisciplinary and internal medicine introductory courses [14] followed by seminars held on 4 days a week, including hands-on ultrasound training, weekly ECG seminars, and courses in clinical pharmacology as well as skills-lab training, critical care management, advanced life support and ward-round training [15]. In addition, theoretical and practical learning processes were supported by logbooks [16], a state examination training course [17], and an on-ward supervision program [18].

### Acquisition of data

The study was conducted over a 16 week period on the premises of the Medical Hospital of the University of Heidelberg, Germany. Data acquisition was executed on the final-year students’ assigned ward during the first and the last two weeks of the FYMS’ Internal Medicine rotation. As clinical performance is highly affected by contextual variables [19], we endeavored to standardize the conditions for activity assessment. All activities were performed during regular, on-ward supervision without spectators. Supervisions took place in patient or examination rooms during the morning shift, lasting a maximum of one and a half hours with German speaking, fully conscious, stable, non-critically ill patients able to undergo physical examination.

### Assessment of clinical activities: Checklists

A Rollei Movieline SD-23 (Rollei GmbH & Co. KG, Hamburg, Germany) camera was used to film clinical performance. All videos were digitally processed and the playing sequence was randomized to forego sequential conclusions. Two blinded, independent video assessors (specially trained physicians and medical educators as well as co-authors of this study) evaluated the students’ performance using four well-established, specific binary checklists [20] for each clinical activity based on faculty standards for history taking, case presentation, IV cannulation [21], and physical examination [22]. Checklist item numbers ranged from 20 (case presentation) to 40 (physical examination).

### Assessment of clinical activities: Global rating

Additionally, eight items (items 2–5, 7–10) from the Integrated Procedural Protocol Instrument (IPPI), as proposed by Kneebone et al. [23], were used to globally assess physical examination and IV cannulation skills. Furthermore, we included two supplementary items to evaluate the completeness and structuring of the procedure. All items were rated via a six-point Likert scale (1 (*strongly agree*), 2 *(mainly agree)*, 3 *(tend to agree)*, 4 *(partially agree)*, 5 *(tend to disagree)*, 6 *(strongly disagree))*.

We assessed case presentation with the Handoff CEX Tool [24, 25], comprising six main domains: *setting, organization, communication, content, judgment,* and *professionalism*. Each domain is scored on a one to nine-point scale, including descriptive anchors at high and low ends of performance to orientate the evaluator. For further guidance, the scale was divided into three overarching sections: 1) unsatisfactory (score 1–3), 2) satisfactory (score 4–6), and 3) superior (score 7–9).

To evaluate history taking, a four item global communication rating scale [26] was used to assess verbal and non-verbal communication and empathy via a six-point Likert scale (1 (*strongly agree*), 2 *(mainly agree)*, 3 *(tend to agree)*, 4 *(partially agree)*, 5 *(tend to disagree)*, 6 *(strongly disagree))*.

### Assessment of clinical competence

Using a model by Lund et al. [27], the overall performance level was evaluated based on the students’ compliance with the raters’ expectations and the level of supervision required via a five-point scale: level 1: below expectations, continuous supervision required; level 2: below expectations, student shows basic skills, supervision required; level 3: meets expectations, sufficient skills under supervision, intermittent supervision required; level 4: above expectations, ready for unsupervised execution; level 5: exceeds expectations, capability to supervise others. In a final step, the overall performance level for each activity was condensed into two overarching categories: 1) “competent” (rated as level ‘5’, ‘4’, and ‘3’) and 2) “incompetent” (rated as level ‘2’ and ‘1’).

### Ethics

The study was conducted according to the Declaration of Helsinki (64th WMA General Assembly, Fortaleza, Brazil, October 2013). Ethics approval was granted by the ethic committee of the University of Heidelberg (S-376/2009). Study participation was voluntary. All students and patients were adequately informed about the study’s purpose and granted anonymity and confidentiality regarding their data. We obtained written informed consent from all participants prior to study participation. Students’ refusal to participate had no impact on subsequent evaluations or other assessments in the curriculum. Patients were advised that they could refuse to participate without having to provide a reason or fear negative effects.

### Statistical analysis

The software package SPSS 20 (Statsoft, Inc., Tulsa, OK, USA) was used for statistical analysis. Data are presented as means ± standard deviation (SD) or as absolute numbers and percentages. Wilcoxon signed rank tests were used for ordinal data (global rating), Bonferroni-Holm correction for multiple comparisons, and paired Student t-tests for interval data (checklist rating, IPPI, CEX) to compare video assessors’ judgments between the start and end of FYMS’ clinical rotation. For the two video assessors, inter-rater reliability was calculated based on Spearman correlation coefficients. Differences in global rating, based on competence-status, were calculated with chi-squared tests. For the explorative assessment of correlations between checklist and global rating scores, Spearman rank correlation coefficients were calculated. A *p*-value < 0.05 was considered to be statistically significant.

## Results

### Participants

All 24 students recruited for the study were FYMS. The mean age was 25.5 years (23; 29), and 62.5% were female participants. Baseline data is shown in Table 1.

**Table 1:** FYMS (*n* = 24) self-assessed job preparedness as well as self-estimated frequency of prior task performance in different training settings (mean, SD)

Clinical activity	Supervised settings ^a^	Unsupervised settings	Job preparedness ^b^
History taking	13.50 (6.65)	64.83 (54.80)	4.46 (.51)
Physical examination	12.58 (6.80)	68.79 (52.75)	4.25 (.61)
Case presentation	3.67 (3.09)	24.96 (41.19)	3.46 (.59)
IV cannulation	5.50 (4.34)	82.92 (67.85)	4.21 (.93)

^a^e.g. skills-lab training, supervised on-ward performance

^b^evaluated by agreement with the statement Concerning [activity named here] I feel well prepared for the job as a medical doctor on a Likert-scale from 1 (not true at all) to 5 (very true)

### Patient sample

Participating patient’s mean age was 59 years (range 27–89) with 32.2% females. 90.3% of the admissions were elective, 9.7% were emergency hospitalizations. In the majority of cases, disease patterns derived from cardiology (29.2%), gastroenterology (17.6%), and hematology/oncology (14.8%) as well as from endocrinology (10.8%) and psychosomatics (10.0%).

### Video rating

#### Checklist and global ratings

Table 2 depicts FYMS’ scores in checklist as well as global and competence ratings at the start and end of rotation. Checklist ratings varied widely but improved significantly for history taking and case presentation. Lowest scores were yielded for case presentation with 39% at t1 and 46% at t2, followed by history taking with 48% at t1 and 58% at t2, and physical examination 55% at t1 and 59% at t2. As predicted, the highest scores were reached for IV cannulation with 81% at t1 and 83% at t2 (s. Table 2).

**Table 2:** FYMS (n = 24) checklist and global rating scores for clinical activities at the start (t1) and end (t2) of Internal Medicine rotation (mean, SD); *p*-values for paired t-tests / Wilcoxon signed rank tests; the category “Competent students” indicates the percentage of students whose performance was rated with level 3 or higher; Pearson’s Χ^2^

Clinical Activity	Assessment	t1 M (SD)	t2 M (SD)	Sig.^d^
History taking				
	Checklist rating [%]	48 (8)	58 (09)	<.001
	GCR ^c^ [1–6]	3.88 (.96)	4.50 (.38)	.004
	Clinical competence [1–5]	2.43 (.71)	3.04 (.53)	.002
	Competent students [%]	33	66	.004
Physical examination				
	Checklist rating [%]	55 (11)	59 (11)	.135
	IPPI^a^ rating [1–6]	4.03 (.54)	4.40 (.35)	.008
	Clinical competence [1–5]	2.40 (.49)	2.96 (.51)	<.001
	Competent students [%]	21	29	.505
Case presentation				
	Checklist rating [%]	39 (14)	46 (14)	.002
	Handoff CEX^b^ [1–9]	3.71 (.89)	4.82 (.81)	<.001
	Clinical competence [1–5]	1.65 (.63)	2.19 (.66)	.001
	Competent students [%]	8	29	.065
IV cannulation				
	Checklist rating [%]	81 (9)	83 (9)	.25
	IPPI^a^ rating [1–6]	4.34 (.65)	4.20 (.59)	.213
	Clinical competence [1–5]	3.19 (.69)	3.60 (.85)	.03
	Competent students [%]	80	87	.6

^a^Integrated Procedural Protocol Instrument

^b^Clinical Examination Exercise

^c^Global communication rating

^d^α = 0,05

During their rotation, students improved significantly in all four activities with regard to the global clinical competence ratings. However, the results of the overall ratings show that the students’ level of performance was not deemed sufficient for unsupervised practice in most cases. Furthermore, the analysis of average scores revealed that student’s overall performances differed significantly.

### Inter-rater-reliability of used instruments

As shown in Table 3, inter-rater reliability proved to be high for case presentation and IV cannulation, while the evaluation of history taking and physical examination performances produced low inter-rater reliabilities in all applied instruments.

**Table 3:** Inter-rater reliabilities (IRR) based on Spearman’s rank correlations (rs) and p-values for checklist as well as global rating scores in the four clinical activities

Clinical Activity	Assessment	IRR rs	p
History taking	Checklist rating [%]	.34	.10
	GCR ^c^ [1–6]	.45	.03
Physical examination	Checklist rating [%]	.57	.003
	IPPI^a^ rating [1–6]	.44	.03
Case presentation	Checklist rating [%]	.70	<.001
	Handoff CEX^b^ [1–9]	.64	.001
IV cannulation	Checklist rating [%]	.83	<.001
	IPPI^a^ rating [1–6]	.88	<.001

^a^Integrated Procedural Protocol Instrument

^b^Clinical Examination Exercise

^c^Global communication rating

#### Correlation between instruments

Table 4 shows correlation coefficients between checklists, global rating scores, and assessed clinical competence. Correlations for checklists were high in all four activities, while global ratings only yielded high correlations for history taking, case presentation, and IV cannulation.

**Table 4:** Correlation coefficients between checklist, global rating scores, and assessed clinical competence in *n* = 24 FYMS across both measurement points; Spearman’s correlation coefficient (rs) and p-value

Clinical Activity	Assessment method	Correlation with assessed clinical competence # [1–5]	
		rs	p
History taking	checklist rating [%]	.57	.004
	GCR ^c^ [1–6]	.59	.002
Physical examination	checklist rating [%]	.73	<.001
	IPPI^a^ rating [1–6]	.19	.38
Case presentation	checklist rating [%]	.71	<.001
	Handoff CEX^b^ [1–9]	.70	<.001
IV cannulation	checklist rating [%]	.85	<.001
	IPPI^a^ rating [1–6]	.84	<.001

^a^Integrated Procedural Protocol Instrument

^b^Clinical Examination Exercise

^c^Global communication rating

#### Assessment of clinical competence

Lowest mean scores were yielded for case presentation (1.65 (SD .63) at t1 and 2.19 (SD .66) at t2). Moderate scores were achieved for physical examination (2.40 (SD .49) at t1 and 2.96 (SD .51) at t2), history taking (2.43 (SD .71) at t1 and 3.04 (SD .53) at t2), and IV cannulation (3.19 (SD .69) at t1 and 3.60 (SD .85) at t2). While most students were rated as competent for the activity IV cannulation (80% at t1 and 87% at t2), only a minority performed case presentation sufficiently (8% at t1 and 29% at t2). In addition, low percentages were reached in physical examination (21% at t1 and 29% at t2) and average percentages were achieved in history taking (33% at t1 and 66% at t2). Chi-squared tests showed significant changes in the percentage of competent students for history taking and change tendencies for case presentation (s. Table 2).

## Discussion

To our best knowledge, this is the first study to (1) descriptively assess the status of FYMS’ objective competencies in four highly relevant clinical activities in a work-place scenario, (2) to examine changes across the course of their 16-week clinical Internal Medicine rotation, and (3) to gain first insight in FYMS’ clinical competence performance level. The study’s main findings suggest that FYMS display deficits when performing clinical, on-ward activities resulting in insufficient preparedness for clinical duty. Although performance generally improved during their sixteen week Internal Medicine rotation, students’ performance levels seemed to be especially low in tasks that were infrequently practiced in clinical settings in preceding medical training.

Despite students largely professing high levels of confidence in regard to self-perceived job preparedness [28], there is evidence for deficits even in their basic clinical skill performance [29, 30]. In accordance with the existing literature, our study revealed that the evaluated FYMS were far from being sufficiently prepared for unsupervised practice in central clinical activities, such as history taking [30], physical examination, and case presentation. Although an integral part of daily ward rounds and clinical practice [29], students failed to achieve more than half of the checklist points for two of the four activities in the beginning of their clinical rotation and still failed to do so for one of the four activities (case presentation) at the end of their clinical rotation. Our results indicate that students only seem to be adequately prepared for i.v.-cannulation at the start of clinical practice.

The absence of improvement in IV cannulation, might be explained by the high percentage of competence students displayed in this task (80%) and by the fact that this activity is frequently trained and supervised in specific medical training settings (skills-lab [31], OSCE [32]). In line with this ceiling effect hypothesis, students showed no significant improvement in the used stepwise assessment measures (checklists) or in the professionalism and quality of execution (IPPI) evaluations of IV cannulation task performance.

Although students have repeatedly practiced patient history taking during specific medical training with standardized patients [33], our results suggest that they still tend to benefit from on-ward medical education to improve their performance levels in clinical competence (competence ratings), procedural accuracy, and the number of correctly performed sub-steps (checklist-ratings) as well as with respect to empathy, verbal, and non-verbal communication abilities (global communication rating).

Professional case presentation training opportunities are rare during clinical routine. Improvement in case presentation was seen for procedural accuracy (checklist-ratings) as well as for form and content (Handoff CEX), possibly leading to a better overall competency rating. However, as mentioned above, students still failed to achieve more than half of the checklist points in this activity at the end of their Internal Medicine rotation.

Regarding the competence in physical examination, the students improved in procedural accuracy (IPPI) but, at the same time, failed to show any significant positive change in the number of correctly performed sub-steps (checklist) or in perceived competence (competent students in %). This might be explained by the fact that the students had the opportunity to practice this essential medical skill between t1 and t2 on the wards (on their own), but mainly did so without any professional supervision [8].

Although it is important to acknowledge that students did gain valuable experiences in actual working conditions during their rotation, clinical, work-place-based education could develop much higher potential with a more balanced, supervised, and needs-based approach. The lack of supervision is a critical point considering the fact that all of these activities constitute elementary and routinely performed day-to-day skills with high relevance for diagnosis, clinical decision making, and, ultimately, treatment plans. Ensuring high performance and professionalism in these basic clinical competencies is indispensable for patient-safety [34].

Further analysis of our data revealed that better objective performance was more pronounced in clinical skills with a higher estimated number of preceding performances (IV cannulation) [8], supporting models advocating deliberate practice and giving emphasis to the importance of repeated, reflective practice [35].

Video assessors’ ratings produced low to moderate inter-rater reliabilities for history taking and physical examinations, and higher inter-rater reliabilities for case presentation and IV cannulation. These results suggest that more observed cases need to be evaluated for these tasks. However, all of the three performance assessment tools reached high inter-rater-reliabilities for case presentation and IV cannulation, with global rating scales achieving higher descriptive values in inter-rater reliabilities for history taking skills compared to checklist ratings and vice versa for physical examination skills. This is in line with the existing literature and confirms that global ratings constitute a more summative measure. They are superior in measuring higher levels of clinical competence, expertise, and professionalism [26, 36], while checklists allow for a more standardized and reliable evaluation of students’ technical performance [37].

Within the four assessed clinical procedures, the applied instruments showed good correlations with assessed clinical competence levels. However, the global rating measure (IPPI) correlated poorly with the assessed clinical competence level of the physical examination. From our experience, this might be explained by three items: namely, item 3 (patient needs assessment), 6 (asepsis maintenance), and 8 (explanation of follow-up care) which are less suitable for on-ward patient care and therefore were not completed in our study in most cases. Vice versa, it is possible that the perceived level of competence is not adequately reflected by the items used in the IPPI.

In summary, when it comes to on-ward training, there are three basic principles in medical education regarding deliberate practice [35]: 1) “the more you practice, the better you get”, 2) “you can only improve activities you do”, and 3) “you can only learn what is taught”. Therefore, to ensure efficient and well-balanced practical education, laissez-faire is not enough. Active efforts towards shaping the form and content of on-ward medical training are imperative. With the objective of ensuring teachable learning opportunities, supporting repeated, reflective practice and addressing observed FYMS’ deficits, a few innovative models have begun to redesign final-year medical education [38, 39]. Additionally, introductory courses [14], on-ward clinical supervision programs [40], and training wards with supervised treatment of real patients have been established [41]. In light of the fact that these approaches are not only costly but also require considerable human resources and expertise, there is an urgent need for innovative models and controlled trials to justify on-ward programs aiming to enhance supervised, independent patient management and structured professional feedback. Moreover, it has been shown recently that active student participation in on-ward patient management enhances doctor-patient interaction compared to standard ward routines [41].

### Limitations

Certain limitations of this study should be noted. Firstly, in consideration of its limited number of participants, our study has to be regarded as a pilot-study. Nevertheless, we were able to provide highly representative data due to minimal drop-out. Secondly, the chosen clinical activities, although being elementary to the daily on-ward routine, can only provide a limited impression of the trainees’ on-ward performance as they are only sequential clippings of their routine. Although multiple testing was not corrected for in the light of the pilot study sample size, results give first important insight in FYMS’ clinical performance. A phenomenon which might have implications for all forms of observational research must not go unmentioned, namely, the inclination to adapt a certain behavior due to the awareness of being studied (Hawthorne effect [42]). Regarding the self-estimated frequencies of the observed clinical activities prior to the study, it is important to understand that it is almost impossible to provide an accurate estimation of these numbers. However, even if the absolute numbers are not correct, it can be assumed that the students are able to classify the frequencies relative to each other.

Finally, it is important to underline that the generalizability of data is limited as it may be difficult to extrapolate findings from a single-center study collected in a single hospital and medical discipline to other areas and faculties. It must be noted that our faculty may offer a more extensive and sophisticated medical curriculum to accompany the final year on-ward training than other hospitals. However, our own observations, as well as the exchange with other medical educators in Germany, have led us to believe that the described structures (and resulting deficits) are largely comparable to those of other German university hospitals.

Nevertheless, the study is unique and takes a first step towards amplifying the necessity of measuring job preparedness in FYMS. However, in accordance with the existing literature [43], future studies should focus on painting a clearer picture of trainees’ performance levels and activity scope by putting the present study’s findings to proof and observing a broad range of activities across multiple measurement points, in varying settings, and with different trainees.

## Conclusions

We were able to show that students’ performance levels differ significantly based on the frequency and complexity of a clinical activity. However, their task performance was not rated as sufficient for independent practice in most cases, resulting in insufficient preparedness for their future jobs. In fact, students even failed to achieve more than half of the checklist points for two of the four activities in the beginning of their clinical rotation and still failed to do so for one of the four activities (case presentation) at the end of their clinical rotation. To adequately prepare students for clinical demands, they need balanced and comprehensive on-ward training as well as sufficient on-ward supervision. In order to secure high quality health-care and patient as well as physician safety, only joint health care professional efforts can seek to improve this situation in future.

## References

[1] Lempp H, Seabrook M, Cochrane M, Rees J (2005). The transition from medical student to doctor: perceptions of final year students and preregistration house officers related to expected learning outcomes. Int J Clin Pract.

[2] Daelmans HE, Hoogenboom RJ, Donker AJ, Scherpbier AJ, Stehouwer CD, van der Vleuten CP (2004). Effectiveness of clinical rotations as a learning environment for achieving competences. Med Teach.

[3] Howley LD, Wilson WG (2004). Direct observation of students during clerkship rotations: a multiyear descriptive study. Acad Med.

[4] Remmen R, Denekens J, Scherpbier A, Hermann I, van der Vleuten C, Royen PV, Bossaert L (2000). An evaluation study of the didactic quality of clerkships. Med Educ.

[5] Van Der Hem-Stokroos HH, Scherpbier AJ, Van Der Vleuten CP, De Vries H, Haarman HJ (2001). How effective is a clerkship as a learning environment?. Med Teach.

[6] Van Der Vleuten CPM, Scherpbier AJJA, Dolmans DHJM, Schuwirth LWT, Verwijnen GM, Wolfhagen HAP (2000). Clerkship assessment assessed. Med Teach.

[7] Sanazaro PJ (1965). Research in medical education: exploratory analysis of a Blackbox. Ann N Y Acad Sci.

[8] Bugaj Till Johannes, Schmid Carolin, Koechel Ansgar, Stiepak Jan, Groener Jan B., Herzog Wolfgang, Nikendei Christoph (2017). Shedding light into the black box: A prospective longitudinal study identifying the CanMEDS roles of final year medical students’ on-ward activities. Medical Teacher.

[9] Bleakley A (2006). Broadening conceptions of learning in medical education: the message from teamworking. Med Educ.

[10] Hannon FB (2000). A national medical education needs’ assessment of interns and the development of an intern education and training programme. Med Educ.

[11] Haney EM, Nicolaidis C, Hunter A, Chan BK, Cooney TG, Bowen JL (2006). Relationship between resident workload and self-perceived learning on inpatient medicine wards: a longitudinal study. BMC Med Educ.

[12] Postgraduate Medical Education: WFME standards for quality improvement. [http://www.wfme.org/]. Accessed 15 Mar 2018.

[13] Nikendei C, Mennin S, Weyrich P, Kraus B, Zipfel S, Schrauth M, Junger J (2009). Effects of a supplementary final year curriculum on students' clinical reasoning skills as assessed by key-feature examination. Med Teach.

[14] Nikendei C, Kraus B, Schrauth M, Weyrich P, Zipfel S, Junger J (2006). An innovative model for final-year students' skills training course in internal medicine: 'essentials from admission to discharge'. Med Teach.

[15] Nikendei C, Kraus B, Lauber H, Schrauth M, Weyrich P, Zipfel S, Junger J, Briem S (2007). An innovative model for teaching complex clinical procedures: integration of standardised patients into ward round training for final year students. Med Teach.

[16] Kraus B, Jünger J, Schrauth M, Weyrich P, Herzog W, Zipfel S, Nikendei C. Logbooks in clinical use - is there a benefit for the students? An Evaluation among final-year-students in internal medicine. GMS Z Med Ausbild. 2007;24(2):Doc112.

[17] Krautter M, Junger J, Koehl-Hackert N, Nagelmann L, Nikendei C (2012). Evaluation of a structured, longitudinal training program for the preparation for the second state exam (M2) - a quantitative analysis. Zeitschrift fur Evidenz, Fortbildung und Qualitat im Gesundheitswesen.

[18] Eden M, Kohl-Hackert N, Krautter M, Junger J, Nikendei C (2010). An innovative model for the structured on-ward supervision of final year students. Med Teach.

[19] ten Cate O (2006). Trust, competence, and the supervisor's role in postgraduate training. BMJ.

[20] Gallagher AG, O'Sullivan GC, Leonard G, Bunting BP, McGlade KJ (2014). Objective structured assessment of technical skills and checklist scales reliability compared for high stakes assessments. ANZ J Surg.

[21] Jünger J, CN (2005). OSCE Prüfungsvorbereitung Innere Medizin.

[22] Pjontek R, Scheibe F, Tabatabai J, Kadmon M, Nikendei C, Huwendiek S, TS (2013). Heidelberger Standarduntersuchung.

[23] Kneebone R, Nestel D, Yadollahi F, Brown R, Nolan C, Durack J, Brenton H, Moulton C, Archer J, Darzi A (2006). Assessing procedural skills in context: exploring the feasibility of an integrated procedural performance instrument (IPPI). Med Educ.

[24] Horwitz LI, Dombroski J, Murphy TE, Farnan JM, Johnson JK, Arora VM (2013). Validation of a handoff assessment tool: the handoff CEX. J Clin Nurs.

[25] Norcini JJ, Blank LL, Arnold GK, Kimball HR (1995). The mini-CEX (clinical evaluation exercise): a preliminary investigation. Ann Intern Med.

[26] Hodges B, Regehr G, McNaughton N, Tiberius R, Hanson M (1999). OSCE checklists do not capture increasing levels of expertise. Acad Med.

[27] Herrmann-Werner A, Nikendei C, Keifenheim K, Bosse HM, Lund F, Wagner R, Celebi N, Zipfel S, Weyrich P (2013). “Best practice” skills lab training vs. a “see one, do one” approach in undergraduate medical education: an RCT on students’ long-term ability to perform procedural clinical skills. PLoS One.

[28] Draper CE, Louw GJ (2012). Competence for internship: perceptions of final-year medical students. Educ Health (Abingdon).

[29] Nikendei C, Kraus B, Schrauth M, Briem S, Junger J (2008). Ward rounds: how prepared are future doctors?. Med Teach.

[30] Ahmed AM (2002). Deficiencies of history taking among medical students. Saudi Med J.

[31] Bugaj T, Nikendei C. Practical clinical training in skills labs: theory and practice. GMS J Med Educ. 2016;33(4):Doc63. 10.3205/zma001062 PMC5003146 27579363

[32] Jünger J, Schäfer S, Roth C, Schellberg D, Friedman Ben-David M, Nikendei C (2005). Effects of basic clinical skills training on objective structured clinical examination performance. Med Educ.

[33] Schultz J-H, Schönemann J, Lauber H, Nikendei C, Herzog W, Jünger J (2007). Einsatz von Simulationspatienten im Kommunikations- und Interaktionstraining für Medizinerinnen und Mediziner (Medi-KIT): Bedarfsanalyse — Training — Perspektiven. Gruppe Interaktion Organisation Zeitschrift für Angewandte Organisationspsychologie (GIO).

[34] Reilly BM (2003). Physical examination in the care of medical inpatients: an observational study. Lancet.

[35] Ericsson KA, Krampe RT, Tesch-Römer C (1993). The role of deliberate practice in the acquisition of expert performance. Psychol Rev.

[36] Newble D (2004). Techniques for measuring clinical competence: objective structured clinical examinations. Med Educ.

[37] Regehr G, MacRae H, Reznick RK, Szalay D (1998). Comparing the psychometric properties of checklists and global rating scales for assessing performance on an OSCE-format examination. Acad Med.

[38] van den Akker M, Dornan T, Scherpbier A, oude Egbrink MG, Snoeckx LH (2012). Easing the transition: the final year of medical education at Maastricht University. Zeitschrift fur Evidenz, Fortbildung und Qualitat im Gesundheitswesen.

[39] Reddy ST, Chao J, Carter JL, Drucker R, Katz NT, Nesbit R, Roman B, Wallenstein J, Beck GL (2014). Alliance for clinical education perspective paper: recommendations for redesigning the "final year" of medical school. Teach Learn Med.

[40] Krautter M, Koehl-Hackert N, Nagelmann L, Junger J, Norcini J, Tekian A, Nikendei C (2014). Improving ward round skills. Med Teach.

[41] Scheffer C, Tauschel D, Neumann M, Lutz G, Valk-Draad M, Edelhauser F (2013). Active student participation may enhance patient centeredness: patients' assessments of the clinical education ward for integrative medicine. Evid Based Complement Alternat Med.

[42] McCambridge J, Witton J, Elbourne DR (2014). Systematic review of the Hawthorne effect: new concepts are needed to study research participation effects. J Clin Epidemiol.

[43] Pennington N, RH, Klein GA, Orasanu J, Calderwood R, CE Z (1993). A theory of explanation-based decision making. Decision Making in Action: Models and Methods.

